# Discovery of a low-latitude ionospheric trough associated with the inner radiation belt

**DOI:** 10.1038/s41598-021-87356-y

**Published:** 2021-04-07

**Authors:** A. T. Karpachev

**Affiliations:** Pushkov Institute of Terrestrial Magnetism, Ionosphere and Radio Wave Propagation (IZMIRAN), Moscow, Russia

**Keywords:** Aurora, Magnetospheric physics

## Abstract

The dynamics of ionospheric troughs that developed during a great geomagnetic storm on 11–13 April 2001 are studied using measurements of electron density obtained by the CHAMP satellite at an altitude of 410–465 km. Subauroral, mid-latitude and low-latitude troughs were observed at nighttime, sometimes simultaneously. The subauroral trough is usually defined as the main ionospheric trough, whereas the mid-latitude trough is associated with the magnetospheric ring current. It appeared at the beginning of the storm recovery phase around latitudes of 40°–45° GMLat (L = 1.7–2.0) and existed for a long period of time throughout the late recovery phase of the residual ring current at latitudes of 50°–55° GMLat (L ~ 2.4–3.0). For the first time, a low-latitude trough was revealed. It developed at latitudes of 34°–45° GMLat (L = 1.45–2.00) in association with the precipitation of energetic particles from the inner radiation belt.

## Introduction

The dynamics of ionization troughs are an essential manifestation of magnetospheric storms in the ionosphere^1^. The ionization trough is usually understood as the main ionospheric trough (MIT); however, during the storm recovery phase, usually at night, another trough, the so-called ring ionospheric trough (RIT) is formed. This second trough is associated with the process of hot ions precipitation from the magnetospheric ring current during its decay^2–4^. The RIT was first separated from the MIT as a distinct structure using Kosmos-1809 data^4^ and has been studied in detail using data from the Interkosmos-19, Kosmos-900 and CHAMP satellites^2,5–8^. Paradoxically, the mechanism of formation of the RIT had already been developed in the 1970s as a means to explain the stable auroral red arc (SAR arc)^9,10^ and was later refined to explain the SAR arc related ionospheric trough^3^. When a severe storm reaches its maximum intensity, the MIT appears at extremely low latitudes. According to data from the Millstone Hill radar for 18 LT during the magnetic storm on 8 February 1986, the trough was recorded at a latitude of 45° GMLat^11^. Since the RIT is observed equatorward of the MIT, it can appear at lower latitudes, approximately 40° GMLat. However, in some cases, the ionospheric trough can form a joint, inseparable structure between the MIT and RIT. As the storm recovery phase develops, the MIT returns to quiet latitudes of > 60° GMLat and the RIT can be observed for long periods of time at the typical latitudes of the residual magnetospheric ring current, i.e. 54°–56°. Additionally, the analysis of CHAMP satellite data from strong storms events has shown the existence of a third trough at very low latitudes, i.e. approximately 35°–45°. These latitudes correspond to the inner radiation belt (IRB) L = 1.5–2.0. Sometimes, all three troughs are observed simultaneously. In this paper, an analysis of the dynamics of all three troughs, i.e. the MIT, RIT and low-latitude trough (LLT), is presented for the magnetospheric storm on 11–13 April 2001.

## Observation data

The analyses presented here are based on data from the CHAMP satellite for high solar activity, obtained in 2000–2002. At high level of solar activity, the magnetospheric storms follow one another continuously. Therefore, the data set for analysis is large, including more than 40 strong geomagnetic storms. This paper presents a detailed analysis of the dynamics of troughs during the severe storm that occurred on 11–13 April 2001. The CHAMP satellite performed in situ measurements of electron density *Ne*. Variations in *Ne* are presented below in terms of plasma frequency *fp*. The CHAMP altitude has changed from 465 km in 2000 to 410 km in 2002, which is close to the height of the *F*2 layer maximum. It revolves in an approximately polar orbit with an inclination of 87.3°. The CHAMP data time resolution of 15 s corresponds to less than 1° of latitude, enabling the position of trough to be determined with a high degree of accuracy. CHAMP satellite data are available on the following website: http://op.gfz-potsdam.de/champ.

### Dynamics of troughs during 11–13 April 2001

**Figure 1:**
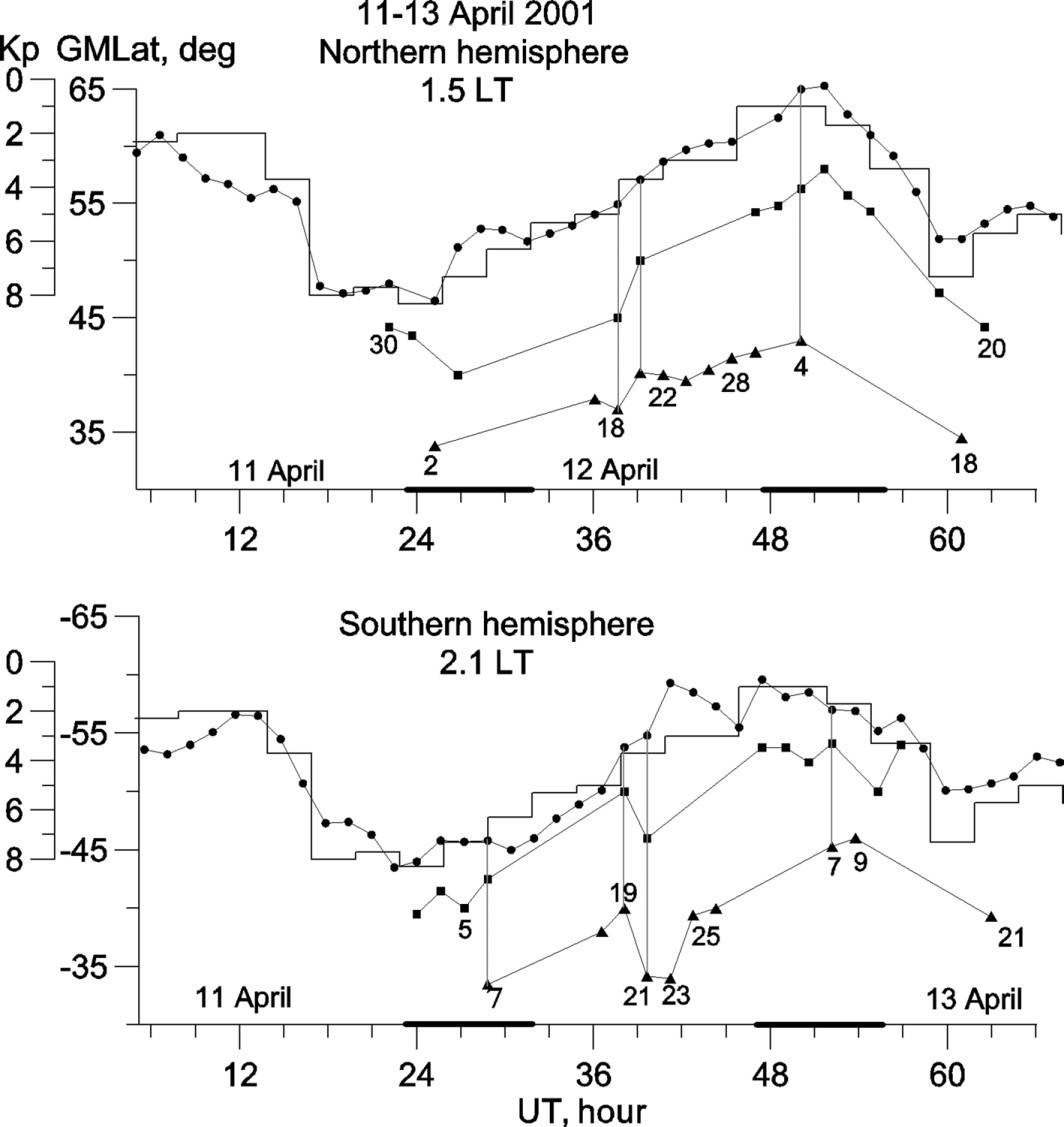
Variations in the position of troughs in the Northern hemisphere for 1.5 LT and the Southern hemisphere for 2.1 LT. The MITs, RITs and LLTs are marked by circles, squares and triangles respectively. The Kp variation is shown by broken line. UT time is counted from 00 UT on April 11. The numbers of characteristic satellite passes are indicated. The longitudes with low geomagnetic field are denoted by bold lines.

The storm on 11–13 April 2001 was extremely strong: Kp index reached 8 + , and Dst was − 271 nT. Figure 1 shows variations in the position of ionospheric troughs for ~ 1.5 LT in the Northern hemisphere and ~ 2.1 LT in the Southern hemisphere. For the convenience of discussion, time is counted from 00 UT on April 11. Variations in the Kp index are related to variations in the position of the MIT (black circles) according to a trough model^12^. This model considers changes in the position of the MIT regarding Kp and local time. A recently revealed dependence on hemisphere^13^ has also been considered. In Fig. 1, the MIT in the Southern hemisphere is shown to be, on average, ~ 3° more equatorward than that in the Northern hemisphere due to differences in local time and hemispherical asymmetry. Variations in the Kp index are shifted to the right because the delay in trough response in both hemispheres is approximately 2.0 h. This delay depends on the growth rate of geomagnetic activity during the main storm phase^12^. Considering this delay, variations in the position of the MIT clearly follow variations in the Kp index. Deviations from the model expectation are primarily related to longitudinal effects. For example, in the Northern hemisphere, during the initial phase of the storm (08–13 UT), the trough turned out at lower latitudes and, during the periods of 26–32 UT and 50–54 UT, it occurred at higher latitudes than expected by the model because of the longitudinal effect. An updated model of the longitudinal effect for both hemispheres and all hours of local time are presented in a previous study^14^. During the period of the storm maximum, the MITs in both hemispheres are located at extremely low latitudes, 44°–46°, corresponding to model expectations.

**Figure 2:**
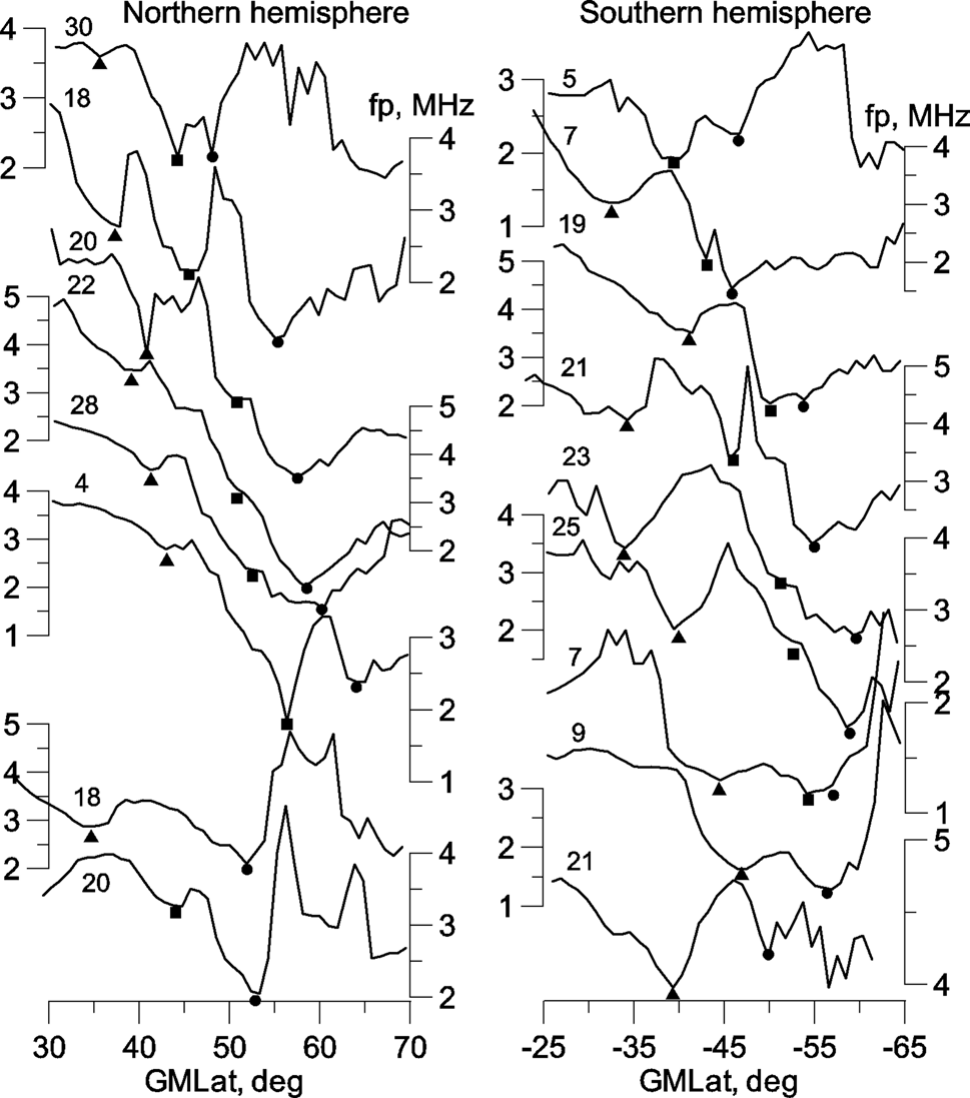
Latitudinal *fp* variations on satellite passes indicated in Fig. 1. The minima of troughs are marked by the corresponding symbols.

The RIT (squares) appears in the Northern hemisphere during the main phase of the storm a few degrees equatorward of the MIT. The RIT forms as a separate trough from the MIT and can be clearly seen in the latitudinal cross-section of *fp* for satellite pass 30 in Fig. 2. During several passes, the RIT was recorded as a weak decrease in electron density (not shown) and was most clearly expressed on pass 18 (12 April). The RIT is manifested as a knee on a steep decline in the electron density on several other passes (20, 22 and 28 in Fig. 2); however, only for pass 20, the pronounced knee is indicated by a triangle as a trough in Fig. 1. Note that, for a very steep slope of electron density, it is difficult to detect a shallow trough. The RIT was again recorded on pass 4 (13 April) as a deep *fp* minimum. It was recorded for the last time in the Northern hemisphere on pass 20 (13 April) after another increase in geomagnetic activity. Figure 1 shows that both the RIT and MIT, generally track variations in the Kp index.

On pass 30 (11 April), the shallow minimum of electron density was observed at a very low latitude of ~ 35° in the Northern hemisphere. This minimum was clearly manifested on pass 2 (12 April), such that it is marked by triangle in Fig. 1 as a trough at a latitude of ~ 34° (L ~ 1.45). However, the LLT was especially pronounced on pass 18, an example of which is shown in Fig. 2. This example is unique in that all three troughs, the MIT, RIT and LLT were strongly pronounced. Cases of simultaneous observation of all three troughs are marked by vertical lines in Fig. 1. On pass 18, the LLT was observed at a latitude of 36°. The formation of three troughs, although not occurring on each satellite pass, suggests the presence of three branches of the trough. They are indicated by thin broken lines in Fig. 1. In the Northern hemisphere, the LLT was also clearly visible on pass 20 and on pass 18 (13 April) following another increase in geomagnetic activity. The LLT was recorded as a shallow minimum of electron density during the remaining passes; this feature can easily be neglected during analysis if the situation is not considered holistically and in terms of its dynamics. Note that the LLT also tracks changes in geomagnetic activity.

In the Southern hemisphere, the situation is similar. All relatively large (2°–3°) deviations in MIT position relative to the model are associated with longitudinal effect^14^. Since the character of the longitudinal effect differs in the Northern and Southern hemispheres, a relatively strong asymmetry in MIT position can observe.

The RIT in the Southern hemisphere appeared at the maximum of the main phase of the storm. On pass 5, it was deeper than the MIT (Fig. 2). During the next passes, the RIT was weakly expressed, including pass 19, where the RIT and MIT formed a joint structure. On pass 21, the RIT appeared as a narrow and deep trough. On passes 23 and 25, an inflection in the latitudinal variations of *fp* was observed at the assumed latitude of the RIT; however, these cases are not marked as troughs in Fig. 1. On pass 7 (13 April), the deep RIT seems to mask the MIT, whose position can be approximated by the base of a steep polar wall. Finally, on pass 11 (April 13), the position of the higher-latitude trough is more likely to correspond to the MIT, as indicated in Fig. 1.

A well-defined LLT in the Southern hemisphere appeared on pass 7 at a latitude of − 33.5°; this also occurred at the beginning of the storm recovery phase. The LLT was more pronounced in the Southern hemisphere than in the Northern hemisphere, particularly in passes 23 and 25 (12 April) and pass 21 (13 April), which occurred after increased geomagnetic activity. In the Southern hemisphere, three simultaneous troughs were most clearly recorded on pass 21 (12 April). The position of the LLT is subject to changes in magnetic activity, as observed in the Northern hemisphere.

It can be seen from Fig. 1 that the RIT and LLT are typically better conjugated than the MIT. The non-conjugacy of the MIT is associated with hemispheric asymmetry and its strong dependence on local time and longitude, as noted above.

**Figure 3:**
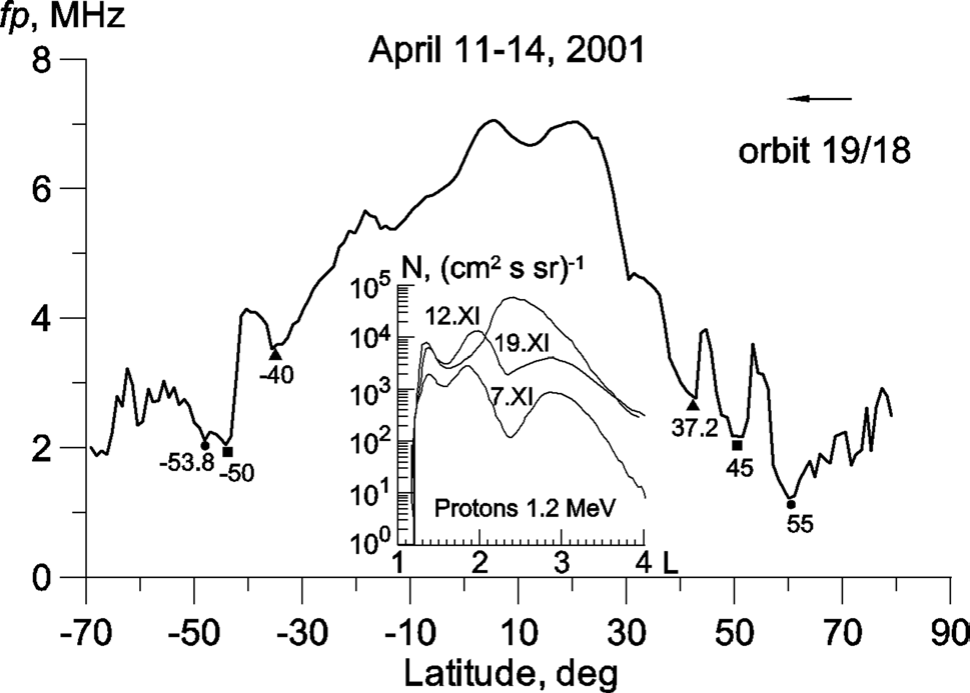
Latitudinal *fp* cross-section on orbits 18 in the Northern hemisphere and 19 in the Southern hemisphere. The inset shows the latitudinal profiles of proton fluxes recorded during and after the double storm on November 7–8 and 9–10, 2004 with Dst -370 nT and -290 nT, respectively. Measurements were carried out onboard the SERVIS-1 satellite at altitude of 500 km^15^. The Figures were made by the Grapher 7.0 program, license WG-052955-1805, installed on the IZMIRAN internal website: https://www.terminator.izmiran.ru.

Figure 3 illustrates the concepts described above. It shows the entire latitudinal *fp* profile in terms of geographical latitude for 18 and 19 passes in the Northern and Southern hemispheres, respectively. In the Northern hemisphere, three extensive troughs (LLT, RIT and MIT) occur at geomagnetic latitudes of 37.2°, 45° and 55°. In the Southern hemisphere, the LLT is located at a latitude of 40°, i.e. the non-conjugation is at 2.8°. A structure with two minima is observed poleward of the LLT. The left minimum at a latitude of − 53.8° corresponds to the MIT, and the discrepancy with the Northern hemisphere in this case is small (1.2°). The right minimum at a latitude of − 50° seems to correspond to the RIT, which is 5° further poleward than in the Northern hemisphere, possibly due to the formation of a wide *fp* peak at latitudes equatorward of the LLT. The inset in Fig. 3 shows an example of the measurements of proton fluxes (1.2 meV) with local peaks on the L-shells ~ 1.4 (32.3°) and ~ 1.9 (43.5°). Similar radial profiles were recorded for electrons (1.7 meV). These fluxes can be responsible for observed structures of the low-latitude ionosphere.

## Discussion

During geomagnetic storms, a strong asymmetric ring current forms in the night sector of the magnetosphere^16^. It mainly comprises ions with energies of 10–100 keV. The RIT develops as a result of the precipitation of hot ions from the magnetospheric ring current during its decay at the recovery phase of the storm^3^. This precipitation occurs when hot ions interact with the cold particles of the plasmasphere. During the recovery phase, the plasmasphere fills up and gradually expands. Thus, the decay process is most intense in the outer part of the plasmasphere, i.e. in a relatively narrow latitude belt. The precipitating ions heat the thermosphere, causing a rise in temperature, an increase in recombination and the formation of an ionization trough.

During the great storm (Dst <  − 300 nT) studied herein, the equatorward edge of the ring current reached L ~ 1.7, i.e. a geomagnetic latitude of ~ 40°^17^. This is an extremely low latitude for the RIT during the maximum of the main phase or the beginning of the recovery phase of a storm. As the recovery phase develops, the plasmasphere expands and the equatorward edge of the ring current also moves to the larger L-shells because of its decay. During the late recovery phase, ion precipitation from the region of the residual ring current is observed for a long period of time around L ~ 2.7‒4.0 (52°‒60°)^18^. The RIT is often observed at latitudes of 54°‒56°. Thus, the region of the RIT is located in the latitude belt between 40° and 60° (L ~ 1.7‒4.0).

The magnetospheric ring current is located in a region of quasi-trapped particle population; the lower the height of the mirror point in the atmosphere, the more intense the resulting ion precipitation. The height of the mirror point depends on the magnitude of the geomagnetic field, which, in turn, strongly depends on the longitude. Thus, we can assume that RIT behaviour also depends on longitude. As shown in Fig. 1, in the Southern hemisphere, bold lines indicate longitudes of 270‒360‒45° with low geomagnetic field magnitudes. The RIT formed mainly at longitudes for which the geomagnetic field was weak. It appeared during the storm maximum at a latitude of ~ 40° and was observed for a long period of time at a latitude of ~ 54° during the late stage of the storm recovery phase. In the Northern hemisphere, longitudes with reduced geomagnetic field magnitudes are almost the same as those in the Southern hemisphere. However, in terms of their absolute values, they are much larger than those in the Southern hemisphere. Thus, since they oscillate along the magnetic field line and drift around the Earth, all particles should be precipitated at longitudes with low geomagnetic field magnitudes in the Southern hemisphere^19^. However, as seen in Fig. 1, the RIT in the Northern hemisphere was most frequently recorded in the period of 47–55 UT, at longitudes marked with a bold line. In this regard, it is notable that Berg^19^ found that the intensity of proton fluxes in the Northern hemisphere is not directly related to local longitudes of observation. Additionally, the longitudinal effect decreased sharply when the geomagnetic activity was increased.

Latitudes between 34° and 45° (L ~ 1.45‒2.00) at which the LLT was observed, correspond to the region of the IRB. This region is usually populated by trapped protons with energies of 20‒500 meV and occupies the region up to L ~ 2, with its maximum at L = 1.5. During magnetic storms, the fluxes of energetic protons and electrons increase sharply (by 1–3 orders of magnitude), apparently due to radial diffusion from higher L-shells^20–23^. These increases are also observed during the storm recovery phase. The latitudinal cross-section of energetic particle fluxes during storms exhibits a complex structure comprising several peaks. During various storms, the KORONAS, SERVIS-1, ACTIVE satellites and MIR station recorded peaks in proton and electron fluxes at L ~ 1.1, 1.4, 1.5, 1.7, 1.8, 1.9, 2.1 and 2.2^15,22,24,25^. These peaks are quasi-stationary in terms of time but change their latitudinal position. An example of the observation of such peaks at L ~ 1.4 and 1.9 was shown in the inset in Fig. 3. Fluxes of trapped particles are associated with very intense precipitation of energetic particles during great geomagnetic storms^26–28^.

Quasi-trapped energetic particles precipitate into the ionosphere as a result of pitch-angular diffusion caused by recharge on neutral hydrogen, scattering on magnetic field irregularities and ion-cyclotron waves^23,29^. Besides direct ionization, enhanced particle precipitation causes an increase in the ionospheric conductivity at the heights of the E layer, growth of the conductivity gradients and, subsequently, the generation of strong local electric fields that can produce strong upward/downward drifts of highly ionized plasma^21,30,31^. In case of sudden changes in the Bz IMF, rapid penetration of the interplanetary electric field has also been observed at low latitudes^32^.

Depending on the direction of the electric field, the drift occurs either upwards or downwards. This can lead to either an increase or decrease in ionospheric plasma density. Ion density enhancements in the topside low-latitude ionosphere during the Bastille storm on 15 and 16 July 2000 and the Halloween storms of 29–31 October 2003 were recorded using data from the ROCSAT-1/IPEI experiment^21^. Prominent ion density enhancements demonstrate similar temporal dynamics in both the sunlit and night-side hemispheres. Ion density increases dramatically (up to two orders of magnitude) during geomagnetic storms. These density enhancements are mostly localized within the region of the South Atlantic Anomaly (SAA), which is characterized by very intense fluxes of energetic particles. These fluxes were investigated using SAMPEX/LEICA data on > 0.6 meV electrons and > 0.8 meV protons at approximately 600 km altitude. During the studied magnetic storms, energetic particle fluxes in the vicinity of the SAA region were found to increase more than by three orders of magnitude.

An analysis of ion temperature and drift velocity in the SAA region shows that ion enhancements are accompanied by enhanced temperature^33^. Plasma heating leads to an increase in recombination at the heights of the F layer and subsequently to density depletion. Thus, processes occurring at IRB latitudes can lead to the formation of ionization peaks and the development of troughs. A previous study^21^ found sharp increases in the ion density in this region, attributing these features to electron precipitation. In light of this observation, Fig. 1 was carefully analyzed to determine whether the structures on the latitudinal *fp* profiles represent peaks or troughs. The clearly defined structures on passes 18 and 20 in the Northern hemisphere and passes 7, 23 and 25 (i.e. 12 and 21 April and 13 April) in the Southern hemisphere are consistent with ionization troughs.

The mirror points for quasi-trapped particles of the IRB, as well as for the ring current, depend on the magnitude of the geomagnetic field. These values fall to their lowest values in the SAA region. Accordingly, when particles drift around the Earth, their L-shell is rapidly emptied at the longitudes and latitudes of this anomaly. Consequently, the majority of the phenomena associated with the precipitation of energetic particles from the IRB, i.e. increased plasma density, ion temperature and atmospheric glow, are most frequently observed in the SAA region. However, particle precipitation from radiation belt and their related phenomena have also been recorded at other longitudes^24,25,33,34^. Figure 1 illustrates that the most pronounced LLT were observed at longitudes with high magnetic field magnitudes, for example, during passes 19–25 in the Southern hemisphere. Hence, the dependence of the occurrence probability of the RIT and LLT on the longitude needs statistical confirmation. This issue will form the subject of future work.

## Conclusion

The main result of this study is the discovery of the LLT at IRB latitudes. This provides an opportunity to observe all three troughs simultaneously, as noted during pass 18 on 12 April in the Northern hemisphere. In other words, three branches of trough are present: subauroral, mid and low latitude.

The MIT is usually understood as a trough located equatorward of the auroral oval, i.e. it is a subauroral trough by definition^13,35^. This trough tracks, with a certain delay, variations in the Kp index according to the MIT model. Delays in MIT response are determined by the growth rate of geomagnetic activity: higher rates correspond to greater delays^12^. The MIT has been studied frequently, yet certain aspects of this feature remain unclear. For example, it remains unknown why the MIT in the Southern hemisphere is, on average, somewhat more equatorward than that in the Northern hemisphere, as seen in Fig. 1.

The second trough, located equatorward of MIT, is a mid-latitude trough. The RIT characteristics are also quite well-established, as denoted by the series of studies cited in the Introduction. These show that the RIT is formed during the initial stage of the recovery phase of severe storms at latitudes of 40°–45° and, during the late stage of the recovery phase, it tends to occur in the latitudes of the residual ring current, i.e. approximately 54°–56°. The RIT is most commonly observed in the morning, during the recovery phase of even a small disturbance. Separation of the RIT and MIT is quite challenging, particularly when they form a joint structure, as observed during passes 19 (12 April) and 7 (13 April) in the Southern hemisphere.

The third, LLT is located at IRB latitudes of 34°–45°. It appears to form only during severe magnetic storms and has not yet been identified during quiet conditions. The LLT is associated with the precipitation of energetic particles from the IRB. This precipitation increases drastically during a storm, including its recovery phase. There is, thus, a reason to believe that such phenomena lead to an increase in the ionospheric conductivity of layer E and, accordingly, to variations in vertical drift velocity. This, in turn, leads to the redistribution of ionospheric plasma, which can manifest itself as a peak or trough of ionization. Additionally, electric fields of magnetospheric origin and heating of the atmosphere can be affected, leading to increased recombination and plasma depletion. These qualitative assumptions, however, should be supported by quantitative simulations.
